# Clinical features of migraine with aura: a REFORM study

**DOI:** 10.1186/s10194-024-01718-1

**Published:** 2024-02-13

**Authors:** Andreas Vinther Thomsen, Håkan Ashina, Haidar M. Al-Khazali, Kathrine Rose, Rune Häckert Christensen, Faisal Mohammad Amin, Messoud Ashina

**Affiliations:** 1 Department of Neurology, Danish Headache Center, Copenhagen University Hospital - Rigshospitalet, Copenhagen, Denmark; 2 Department of Clinical Medicine, Faculty of Health and Medical Sciences, University of Copenhagen, Copenhagen, Denmark; 3 Department of Brain and Spinal Cord Injury, Copenhagen University Hospital - Rigshospitalet, Copenhagen, Denmark; 4 Department of Anesthesia, Critical Care and Pain Medicine, Beth Israel Deaconess Medical Center, Boston, MA USA; 5 Harvard Medical School, Boston, MA USA; 6 Danish Knowledge Center On Headache Disorders, Copenhagen University Hospital - Rigshospitalet, Copenhagen, Denmark

**Keywords:** Migraine, Migraine with aura, Headache, Observational study

## Abstract

**Background:**

About one-third of persons with migraine experience transient neurologic symptoms, referred to as aura. Despite its widespread prevalence, comprehensive clinical descriptions of migraine with aura remain sparse. Therefore, we aimed to provide an in-depth phenotypic analysis of aura symptoms and characteristics in a cross-sectional study of a large sample of adults diagnosed with migraine with aura.

**Methods:**

Data were extracted from the baseline characteristics of participants in the Registry for Migraine (REFORM) study – a single-center, prospective, longitudinal cohort study. Participants were adults diagnosed with migraine aura, reporting ≥ 4 monthly migraine days in the preceding 3 months. Trained personnel conducted in-person semi-structured interviews, capturing details on the nature, duration, localization, and progression of individual aura symptoms.

**Results:**

Of the 227 enrolled participants with migraine with aura, the mean age was 41.1 years, with a predominant female representation (*n* = 205 [90.3%]). Visual aura was present in 215 (94.7%) participants, somatosensory aura in 81 (35.7%), and speech and/or language aura in 31 (13.7%). A single type of aura was observed in 148 (65.2%) participants, whilst 79 (34.8%) reported multiple aura types. Most participants (*n* = 220 [96.9%]) described their aura symptoms as positive or gradually spreading. Headache in relation to aura was noted by 218 (96.0%) participants, with 177 (80.8%) stating that the onset of aura symptoms preceded the onset of headache.

**Conclusions:**

This study offers a detailed clinical depiction of persons with migraine with aura, who were predominantly enrolled from a tertiary care unit. The findings highlight potential gaps in the available literature on migraine with aura and should bolster clinicians’ acumen in diagnosing migraine with aura in clinical settings.

**Supplementary Information:**

The online version contains supplementary material available at 10.1186/s10194-024-01718-1.

## Introduction

Migraine, a disabling neurologic disorder, affects more than 1 billion persons worldwide [1, 2]. Among those with migraine, about one-third experience migraine with aura (MA), characterized by transient neurologic symptoms [3–8]. Aura typically precedes or accompanies the headache phase of a migraine attack, although it can occur in isolation and, in some cases, exclusively so [9, 10]. The most prevalent manifestation is visual aura, often presenting as fortification spectra and reported by more than 90% of persons with MA [9, 10]. Somatosensory aura, primarily characterized by gradually spreading unilateral paresthesia in the face or arm, occurs less often, affecting about 30% of persons with MA [10]. Furthermore, rare aura manifestations include speech and/or language disturbances (e.g., aphasia), motor weakness, brain stem symptoms (e.g., ataxia, diplopia), and retinal symptoms (e.g., monocular visual disturbances) [10].

While the 3rd edition of the International Classification of Headache Disorders (ICHD-3) [10] provides valuable insights into the clinical features of aura, the available evidence from observational studies often lacks the detail necessary for a thorough understanding. Small sample sizes and the exclusion of specific aura symptoms have limited a comprehensive assessment of aura characteristics in persons with migraine [11–13]. Additionally, differences in study populations (e.g. population- or clinic-based) and methodologies (e.g. retrospective or prospective recording, and the use of structured interviews, diaries or other methods of data collection) render direct comparisons between studies difficult [9, 11, 14, 15]. Addressing this critical gap is paramount to improving disease characterization and enabling accurate diagnoses in clinical practice.

In this study, we aim to provide an in-depth characterization of aura features in a large sample of adult participants diagnosed with migraine with aura. This, in turn, allows us to explore the clinical features of aura in great detail and contribute to a more comprehensive disease characterization. Our findings will thus provide valuable insights for clinical practice and serve as a foundation for further research in this field.

## Methods

The Registry for Migraine (REFORM) study is a prospective, longitudinal cohort study conducted at a single center. It aimed to investigate adult participants with migraine who were scheduled to participate in a phase IV trial (NCT04265755). Detailed information regarding the parent REFORM study has been published elsewhere [16]. The study protocol received approval from the Health Research Ethics Committee of the Capital Region of Denmark, and all participants provided written informed consent before undergoing any study-related assessments or procedures. The study adhered to the principles outlined in the Declaration of Helsinki, including its subsequent revisions [17]. Enrollment spanned from September 2020 to July 2022.

### Participants

Participants were primarily recruited from the outpatient clinic of a tertiary care unit. Eligible individuals were males and females, aged ≥ 18 years, with a documented history of migraine lasting ≥ 12 months, migraine onset prior to age 50, and experiencing an average of ≥ 4 monthly migraine days in the last 3 months before screening [16]. Exclusion criteria included a history of cluster headache or hemiplegic migraine, as well as difficulty in differentiating migraine from other types of headaches. The complete list of inclusion and exclusion criteria can be found elsewhere [16]. This paper focuses on participants who completed the semi-structured interview in the parental REFORM study [16] and reported a lifetime history of migraine with aura according to ICHD-3 [10]. The data reported in the present were extracted from the baseline characteristics of participants in the REFORM study.

### Procedures

Trained personnel conducted a semi-structured interview to collect demographic data, clinical characteristics, and a detailed history of the disease course from all participants. The interview included assessment of the number and characteristics of aura episodes in the past month, past three months, and past year, as well as the total number of lifetime aura episodes, following the ICHD-3 criteria for migraine with aura. Participants who reported multiple types of aura were asked about the temporal relationship between the different aura types. Furthermore, participants were queried about the presence of headache in relation to aura episodes, and detailed information on headache features was obtained. Any uncertainties were resolved through consultation with the senior author (M.A.).

### Outcomes and measures

We investigated presence of headache disorders other than MA as well as monthly migraine days and monthly aura days for all participants. The symptoms and characteristics of aura were defined in accordance with the ICHD-3 criteria and descriptions for *1.2 Migraine with aura* and its subtypes. We used a semi-structured interview to distinguish aura from other manifestations and features of migraine [10]. For each aura symptom reported, we investigated individual symptom characteristics (laterality and duration of symptoms, presence of positive symptoms, gradually spreading symptoms, accompanying headache and number of aura symptoms). For participants reporting more than 1 aura symptom, individual combinations and sequences were recorded. For participants reporting aura with headache, we investigated headache symptoms in detail, including headache characteristics (duration, intensity, laterality, character, aggravation with physical movement), associated symptoms (nausea, photophobia and phonophobia), as well as reported provoking factors, neck pain, dizziness, vertigo, autonomic symptoms, premonitory symptoms and postdromal symptoms. We additionally assessed if headache accompanying aura fulfilled ICHD-3 criteria B-D for *1.1 migraine without aura* [10]*.* Active MA was defined as at least 1 aura episode in the last 12 months.

### Statistical analysis

Continuous variables were summarized as means ± standard deviations (SDs), while categorical data were presented as numbers and percentages. All analyses were carried out using *R* version 4.2.0.

## Results

A total of 751 persons were enrolled in the REFORM study. Of these, 227 participants (30.2%) had a lifetime history of aura and were included the present study (Table 1). The mean age of the participants with MA was 41.1 years (SD, 12.2) and most (90.3%) were female. The mean body mass index was 24.9 kg/m^2^, and participants experienced a mean number of 21.6 (SD, 6.8) monthly headache days. Among the participants with MA, 211 (93.0%) reported co-existing migraine without aura, and 158 (69.6%) met the criteria for chronic migraine. In the past 12 months, 196 (86.0%) participants reported at least 1 episode of aura (i.e., active MA), and the mean number of monthly aura episodes in the last year was 3.8 (SD, 4.9). Moreover, 77 (33.9%) participants experienced episodes of aura without headache, and 8 (3.5%) reported never experiencing headache in relation to their aura episodes. Concomitant use of acute medication was reported by 216 (95.2%) on a mean 11.8 days per month (SD, 6.9), and 119 (52.4%) participants reported concomitant use of medication for migraine prevention.

**Table 1:** Demographics, baseline characteristics, and headache disorders of the study population

All (*N* = 227)		Female (*n* = 205)	Male (*n* = 22)
Age (years)	44.1 (12.2)	44.1 (12.0)	43.7 (14.2)
Height (cm)	170.0 (7.5)	168.8 (6.3)	181.8 (7.2)
Weight (kg)	72.1 (16.8)	71.3 (16.5)	80.4 (17.8)
BMI (kg/m^2^)	24.9 (5.4)	25.0 (5.4)	24.2 (4.8)
Race			
White	226 (99.6)	204 (99.5)	22 (100)
Black	0 (0)	0 (0)	0 (0)
Asian or Pacific Islander	1 (0.4)	1 (0.5)	0 (0)
Occupational status			
Full-time employment or studies	114 (50.2)	104 (50.7)	10 (45.5)
Part-time employment or studies	46.0 (20.3)	44 (21.5)	2 (9.1)
Without employment or studies	21 (9.3)	16 (7.8)	5 (22.7)
Retired	19 (8.4)	17 (8.3)	2 (9.1)
Other	27 (11.9)	24 (11.7)	3 (13.6)
Migraine with aura^a^			
Age of MA onset (years)	24.6 (12.1)	24.9 (12.9)	21.3 (12.8)
Disease duration of MA (years)	19.5 (13.8)	19.2 (13.8)	22.4 (13.8)
Active MA^b^	195 (85.9)	176 (85.9)	19 (86.4)
MA without co-existing MO	16 (7.0)	16 (7.8)	0 (0)
Aura with headache	218 (96.5)	197 (96.6)	21 (95.5)
Aura without headache	77 (33.9)	69 (33.7)	8 (36.4)
Only aura without headache	8 (3.5)	7 (3.4)	1 (4.5)
Chronic migraine^a^	158 (69.6)	142 (69.2)	16 (72.7)
Migraine without aura^a^	211 (93.0)	189 (92.2)	22 (100)
Tension-type headache^a^	30 (13.2)	28 (13.7)	2 (9.1)
Medication overuse headache^a^	81 (35.7)	75 (36.6)	6 (27.3)
Migraine days in last month	14.9 (6.9)	15.1 (6.9)	14 (7.2)
Headache days in last month	21.6 (6.8)	21.6 (6.9)	21.9 (6.7)
Aura episodes in last month	3.8 (5.1)	3.9 (5.3)	3 (3.3)
Monthly auras in last year	3.8 (4.9)	3.8 (5.1)	3.1 (3.3)
Concomitant acute medication	216 (95.2)	197 (96.1)	19 (86.4)
Concomitant migraine prevention	119 (52.4)	109 (53.2)	10 (45.5)
Prior migraine prevention failures			
0	26 (11.5)	24 (11.7)	2 (9.1)
1	10 (4.4)	10 (4.9)	0 (0)
2–4	114 (50.2)	100 (48.8)	14 (63.6)
5 or more	77 (33.9)	71 (34.6)	6 (27.3)

Data are mean (SD) or n (%)

*Abbreviations*: *BMI* Body-mass index, *CM* Chronic migraine, *EM* Episodic migraine, *MA* Migraine with aura, *MO* Migraine without aura, *SD* Standard deviation

^a^Multiple diagnoses are possible. Diagnosis of chronic headache precludes diagnosis of tension-type headache

^b^Active MA defined as at least 1 aura episode in past 12 months

### Aura symptoms

**Fig. 1:**
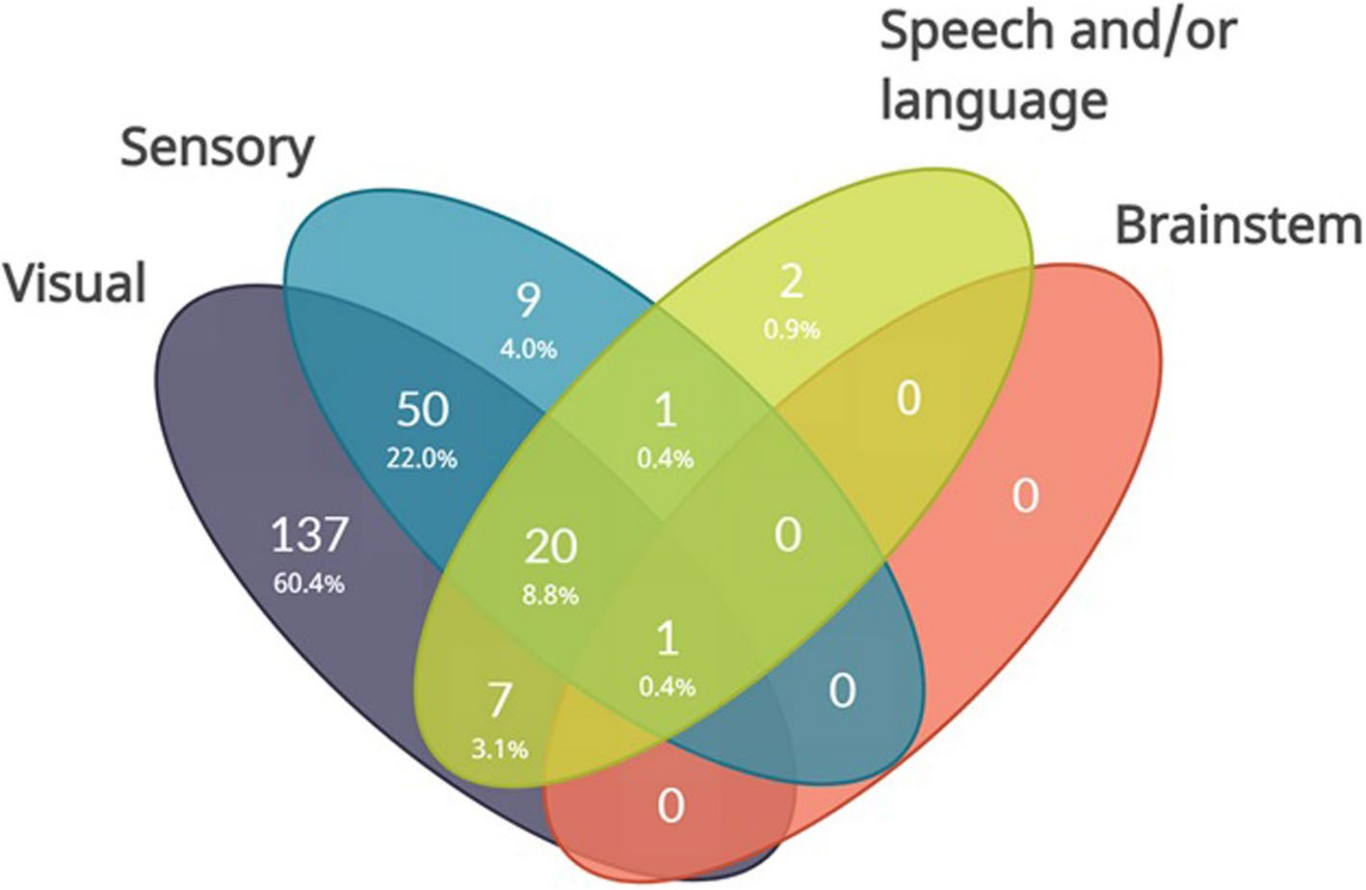
Aura symptom combinations. Venn diagram with all combinations of aura types. Fields contain numbers of participants and corresponding percentages

The presence of different aura symptoms was distributed as follows: visual aura was reported by 215 (94.7%) participants, somatosensory aura by 81 (35.7%) participants, speech and/or language aura by 31 participants (13.7%), and brain stem aura by 1 participant (0.4%), (Table 2). It is worth noting that 148 (65.2%) of 227 participants exclusively experienced 1 type of aura, while the remaining 79 (34.8%) reported multiple types of aura. Figure 1 provides a visual representation of the individual aura types and their combinations.

**Table 2:** Aura symptoms and individual symptom features

	**All (*n* = 227)**	**EM (*n* = 69)**	**CM (*n* = 158)**
**Visual aura**	215 (94.7)	67 (97.1)	148 (93.7)
Positive symptoms	192 (89.3)	63 (94.0)	129 (87.2)
Negative symptoms	127 (59.1)	38 (56.7)	89 (60.1)
Zig-zag pattern	76 (35.3)	22 (32.8)	54 (36.5)
Flickering	151 (70.2)	52 (77.6)	99 (66.9)
Sawtooth edge	30 (14.0)	13 (19.4)	17 (11.5)
Gradually spreading	155 (72.1)	51 (76.1)	104 (70.3)
Central onset^a^	45 (20.9)	11 (16.4)	34 (23.0)
Peripheral onset^a^	109 (50.7)	39 (58.2)	70 (47.3)
Mean duration (min)	49.2 (109.7)	37.6 (35.6)	54.4 (129.8)
Duration < 5 min	8 (3.7)	3 (4.5)	5 (3.4)
Duration 5–60 min	185 (86.0)	59 (88.1)	126 (85.1)
Duration > 60 min	22 (10.2)	5 (7.5)	17 (11.5)
Unilateral symptoms	109 (50.7)	38 (56.7)	71 (48.0)
Sidelocked symptoms^b^	51 (23.7)	17 (25.4)	34 (23.0)
Aura ipsilateral to headache^c^	18 (8.4)	5 (7.5)	13 (8.8)
Aura contralateral to headache^c^	64 (29.8)	22 (32.8)	42 (28.4)
**Somatosensory aura**	81 (35.7)	21 (30.4)	60 (38.0)
Positive symptoms	69 (85.2)	18 (85.7)	51 (85.0)
Negative symptoms	28 (34.6)	7 (33.3)	21 (35.0)
Gradually spreading	58 (71.6)	13 (61.9)	45 (75.0)
Mean duration (min)	136.7 (503.4)	299.5 (934.0)	79.8 (187.4)
Duration < 5 min	2 (2.5)	0	2 (3.3)
Duration 5–60 min	56 (69.1)	13 (61.9)	43 (71.7)
Duration > 60 min	23 (28.4)	8 (38.1)	15 (25.0)
Unilateral symptoms	55 (67.9)	16 (76.2)	39 (65.0)
Sidelocked symptoms^b^	35 (43.2)	11 (52.4)	24 (40.0)
Aura ipsilateral to headache^c^	8 (9.9)	5 (23.8)	3 (5.0)
Aura contralateral to headache^c^	35 (43.2)	8 (38.1)	27 (45.0)
**Speech and/or language aura**	31 (13.7)	11 (15.9)	20 (12.7)
Non-fluent aphasia	23 (74.2)	8 (72.7)	15 (75.0)
Fluent aphasia	5 (16.1)	0	5 (25.0)
Dysarthria	9 (29.0)	5 (45.5)	4 (20.0)
Mean duration (min)	65.7 (109.7)	103 (170.9)	44.2 (42.9)
Duration < 5 min	1 (3.2)	0	1 (5.0)
Duration 5–60 min	24 (77.4)	8 (72.7)	16 (80.0)
Duration > 60 min	5 (16.1)	3 (27.3)	2 (10.0)
**Number of aura symptoms**			
1	148 (65.2)	48 (69.6)	100 (63.3)
2	58 (25.6)	12 (17.4)	46 (29.1)
3	20 (8.8)	9 (13.0)	11 (7.0)
4	1 (0.4)	0	1 (0.6)

Data are mean (SD) or n (%)

*Abbreviations*: *CM* Chronic migraine, *EM* Episodic migraine, *SD* Standard deviation

^a^Includes only participants with gradually spreading symptoms

^b^Includes only participants with unilateral symptoms

^c^Includes only participants with both unilateral aura symptoms and unilateral headache

Among the 79 participants who experienced multiple aura types, 56 (70.9%) reported their aura symptoms occurring successively, while 19 participants (23.9%) experienced a simultaneous onset of aura symptoms. Four participants either could not recall the association or did not experience more than 1 aura type in each episode. Of the 56 participants with successive aura symptoms, visual aura was the initial presenting symptom for 39 (69.6%) participants, somatosensory aura for 13 (23.2%) participants, and speech and/or language aura for 2 (3.6%) participants. One additional participant (1.8%) reported experiencing simultaneous onset of visual and speech and/or language aura followed by somatosensory aura. Furthermore, the sequence of aura symptoms could not be recalled by 1 participant. A detailed representation of individual aura symptom sequences is provided in Table 3.

**Table 3:** Sequence of aura symptoms in participants experiencing ≥ 2 aura symptoms in succession

Participants with successive aura symptoms (*n* = 56^a^)						
**2 symptoms (*n* = 42)**			**3 symptoms (*n* = 13)**			
First symptom	Second symptom	n (%)	First symptom	Second symptom	Third symptom	n (%)
V	S	28 (66.6)	V	S	A	5 (48.5)
S	V	10 (23.8)	V	A	S	2 (15.4)
V	A	2 (4.8)	V	B^b^	S	1 (7.7)
S	A	1 (2.4)	S	A	V	1 (7.7)
A	V	1 (2.4)	S	V	A	1 (7.7)
			A	V	S	1 (7.7)
			V + A^b,c^	S		1 (7.7)
			V	S + A^c,d^		1 (7.7)

*Abbreviations*: *A* Aphasic aura, *B* Brainstem aura, *S* Somatosensory aura, *V* Visual aura

^a^One participant could not recall sequence

^b^The participant with brainstem aura additionally experienced occasional aphasic aura, but not in connection with other aura symptoms

^c^Visual and aphasic aura presented together and were followed by somatosensory aura

^d^Visual aura presented alone and was followed by somatosensory and aphasic aura simultaneously

### Aura characteristics

Among the 227 participants, the most prevalent aura characteristics were the presence of at least 1 positive symptom, reported by 210 participants (92.5%), and the occurrence of aura accompanied or followed by headache within 60 min, reported by 209 participants (92.1%). Furthermore, most participants, 177 (78.0%), reported each aura symptom lasting between 5 to 60 min, and 170 (74.9%) participants experienced the spreading of at least 1 aura symptom for a duration of ≥ 5 min. Of note, 220 (96.9%) participants experienced aura symptoms that were either positive and/or spreading ≥ 5 min. Detailed characteristics for each individual aura symptom can be found in Table 2, and summarized characteristics are provided in Table 4.

**Table 4:** Summary of aura symptom features

All migraine with aura (*n* = 227)	
**Aura symptom feature**	
At least 1 symptom spreads ≥ 5 min	170 (74.9)
Two or more symptoms occur in succession	56 (24.7)
At least 1 symptom is positive	210 (92.5)
Each symptom lasts 5–60 min	177 (78.0)
At least 1 symptom is unilateral	143 (63.0)
Aura is accompanied, or followed within 60 min, by headache	209 (92.1)
At least 1 symptom positive and/or spreading ≥ 5 min	220 (96.9)

Data presented as n (%)

Among the 155 participants who reported gradually spreading visual aura, the onset was observed in the central visual field in 45 (29.0%) participants and in the peripheral visual field in 109 (70.3%) participants. One participant was unable to recall the site of onset. Moreover, unilateral visual aura was reported by 109 (50.7%) of 215 participants and among these, aura symptoms were side-locked in 51 participants. Of the 81 participants with somatosensory aura, the most frequently involved anatomical regions were the hand (*n* = 64 [79.0%]), arm (*n* = 47 [58.0%]), and face (*n* = 39 [48.1%]). Side-locked somatosensory aura was experienced by 35 (43.2%) of 81 participants. We did not find any notable differences in aura symptom characteristics between participants with and without concomitant use of preventative medication for migraine (Supplemental Table S1).

### Headache characteristics

Among the 227 participants with MA, 218 (96.0%) reported experiencing headache in association with their aura and in 181 participants, the headache fulfilled the ICHD-3 [10] criteria for migraine without aura. The most frequent headache features were moderate to severe pain intensity, reported by 215 (98.6%) participants, and pain aggravation with routine physical activity, reported by 182 participants (83.5%) (Table 5). Among the 215 participants with visual aura, 18 (8.4%) reported ipsilateral headache, while 64 (29.8%) experienced contralateral headache. For the 81 participants with somatosensory aura, 8 (9.9%) reported ipsilateral headache and 35 (43.2%) experienced contralateral headache. The remaining participants reported either bilateral headache and/or aura.

**Table 5:** Individual features of headache accompanying aura

All migraine with aura with headache (*n* = 218)	
Pain intensity (NRS 0–10)	8.2 (1.4)
Headache duration untreated or unsuccesfully treated (hours)	46.3 (32.1)
Headache duration < 4 h	4 (1.8)
Headache duration 4–24 h	82 (37.6)
Headache duration 24–72 h	106 (48.6)
Headache duration > 72 h	21 (9.6)
Unilateral headache	180 (82.6)
Headache moderate/severe intensity	215 (98.6)
Headache pulsating/throbbing	166 (76.1)
Headache aggravated by physical activity	182 (83.5)
Nausea/vomiting	195 (89.4)
Photophobia	206 (94.5)
Phonophobia	199 (91.3)
Neck pain	125 (57.3)
Dizziness	86 (39.4)
Vertigo	35 (16.1)
Autonomic symptoms	117 (53.9)
Provoking factors	118 (54.1)
Premonitory symptoms	88 (40.4)
Postdromal symptoms	104 (47.7)
Headache fulfills ICHD-3 criteria for 1.1 Migraine without aura	181 (83.0)

Data are mean (SD) or n (%)

*Abbreviations*: *CM* Chronic migraine, *EM* Episodic Migraine, *ICHD-3* International Classification of Headache Disorders third edition, *NRS* Numeric rating scale

Of 218 participants reporting migraine aura with headache, 177 (80.8%) experienced onset of headache after the onset of aura, with a mean (SD) interval in between of 41.7 (58.1) minutes. In 4 participants, headache onset occurred more than 60 min after their migraine aura. Furthermore, 26 (11.9%) participants reported simultaneous onset of aura and headache, while 11 (5.0%) participants experienced the aura onset after headache onset. Four participants could not recall the timing of the onset.

### Migraine attacks with and without aura

**Fig. 2:**
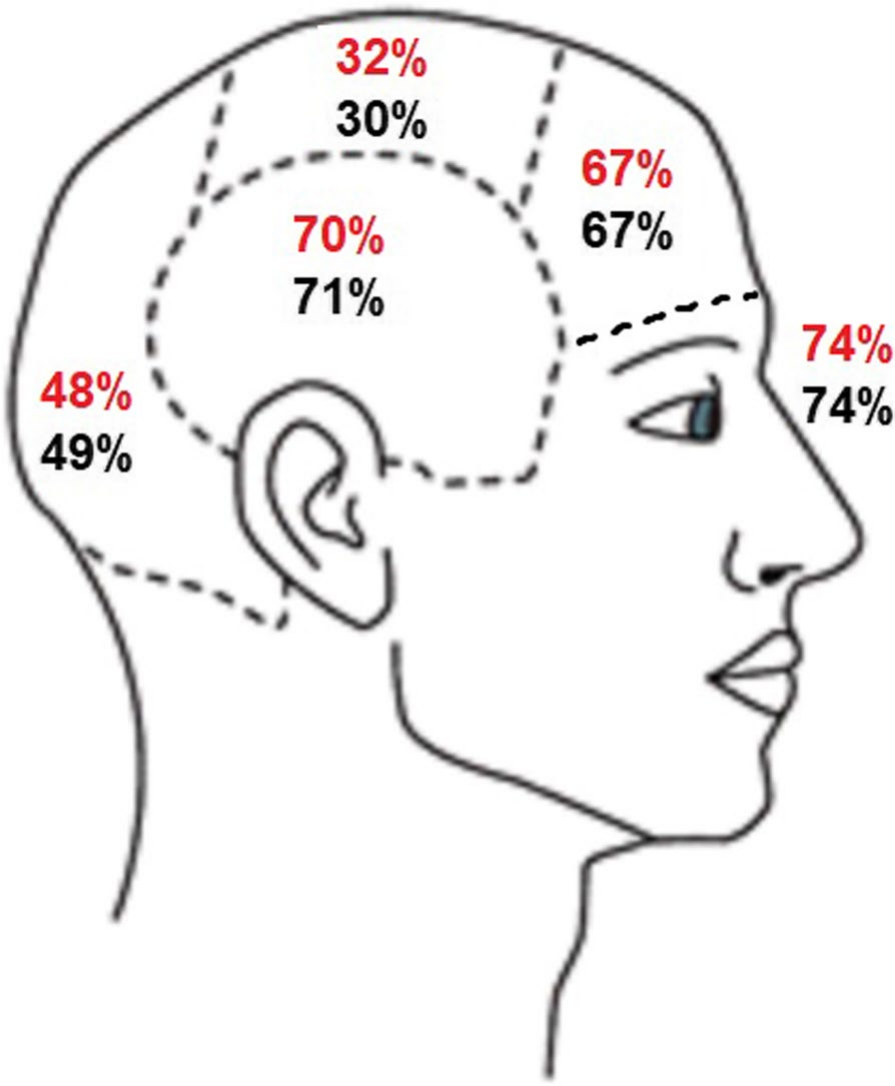
Headache localization in migraine with and without aura. Red numbers are in migraine with aura, black numbers are migraine without aura

Co-existing migraine attacks with and without aura were reported by 202 out of 227 participants (89.0%). The headache features and accompanying symptoms for each type of attack are detailed in Table 6, and the localization of headaches are provided in Fig. 2.

**Table 6:** Comparisons of headache features between episodes with and without aura in participants with co-existing MAWH and MO

	**MAWH and co-existing MO (*n* = 202)**	
	**MAWH**	**MO**
Pain intensity (NRS 0–10)	8.1 (1.5)	7.7 (1.5)
Headache duration untreated or unsuccessfully treated (hours)	46.5 (32.0)	49.9 (32.2)
Unilateral headache	168 (83.2)	166 (82.2)
Headache moderate/severe intensity	199 (98.5)	201 (99.5)
Headache pulsating/throbbing	152 (75.2)	162 (80.2)
Headache aggravated by physical activity	167 (82.7)	189 (93.6)
Nausea/vomiting	179 (88.6)	169 (83.7)
Photophobia	191 (94.6)	194 (96.0)
Phonophobia	186 (92.1)	189 (93.6)
Neck pain	116 (57.4)	131 (64.9)
Dizziness	82 (40.6)	85 (42.1)
Vertigo	30 (14.9)	29 (14.4)
Autonomic symptoms	108 (53.7)	120 (59.4)
Provoking factors	105 (52.0)	188 (93.1)
Premonitory symptoms	81 (40.1)	130 (64.4)
Postdromal symptoms	96 (47.5)	149 (73.8)

Data are mean (SD) or n (%)

*MAWH* Migraine aura with headache, *MO* Migraine without aura, *NRS* Numeric rating scale

## Discussion

In the present study, we meticulously characterized the aura features in a large sample of 227 adults diagnosed with MA. Our findings reveal a high prevalence of visual aura, reported by 215 (94.7%) participants. Less common was somatosensory aura (35.7%) and speech and/or language aura (13.6%), while only 1 participant experienced brainstem aura. These insights align well with prior observational studies and also extend beyond by providing a more granular view of individual aura characteristics [6, 9, 11–13, 18–22]. The implications of this have implications for both clinical practice and further research, as elucidated in the following sections.

### Visual aura

The present findings indicated that visual aura often manifests as gradually spreading, positive symptoms with a duration between 5 and 60 min. Traditionally considered unilateral [10], we found that about half of our participants experienced bilateral visual aura. In addition to this, an interesting observation was that 70.3% of the participants experienced onset of visual aura in the peripheral visual field. This contrasts the typical depiction of visual aura as a fortification spectrum commencing in the central visual field and then gradually spreading to the periphery [10]. However, our observation does accord well with findings from a prospective, observational study, and some neuroimaging evidence demonstrating initiation of CSD in visual area V3A [11, 23].

Another important observation pertains to the duration of visual aura, which was found to be less than 60 min in 89.8% of our participants. Consequently, clinicians should consider secondary etiologies for aura when visual manifestations exceed this duration. Additional clinical evaluation should also extend to cases where aura symptoms occur without visual manifestations, as a mere 5.3% of our MA participants did not experience visual aura. We found that a large proportion of our participants (89.3%) reported positive symptoms, a finding in line with previous findings [9, 12]. A prospective study found a frequency of 67% in 212 recorded aura attacks [11]. This discrepancy highlights the difficulties in comparing findings between available studies due to differences in study populations and methodologies. Compared to some other studies, we investigated a limited number of visual aura symptoms [11, 12, 14, 15]. Variability in specific visual aura features (e.g. shapes, colours, tunnel-vision, and the degree of involvement of the visual field) may be of special interest to researchers in dissecting possible differences in the propagation of CSD between patients.

### Somatosensory aura

Akin to visual aura, our findings revealed that somatosensory aura manifested predominantly with positive, gradually spreading symptoms. These somatosensory disturbances were usually localized to the hand, arm, and face, corroborating previous reports [9]. Unlike visual aura, the duration of somatosensory aura lasted for more than 60 min in about one-third of our participants, with symptoms being reported as unilateral in two-thirds of the cases. These insights bolster the current understanding that non-spreading, negative symptoms should raise clinical suspicion for secondary etiologies, necessitating further diagnostic work-up [24].

### Speech- and/or language aura

Non-fluent aphasia, intrinsically a unilateral symptom [10], emerged as the predominant feature of speech and/or language aura in our study. The duration of these symptoms closely mirrored that of visual aura, with only 16.1% of the participants reporting a duration exceeding 60 min. Another interesting observation was the typical co-existence of speech and/or language aura with visual and/or somatosensory aura. Indeed, our data revealed that only 2 (6.5%) of 31 participants exhibited speech and/or language aura in isolation. Cases presenting as such at the initial clinical evaluation should therefore prompt clinicians to consider secondary etiologies.

### Multiple aura symptoms

About one-third of our participants reported experiencing ≥ 2 distinct aura symptoms, and, consistent with prior studies [6, 9, 11], visual aura was found to often manifest alone, whilst other aura symptoms seldom presented in isolation. Among the 57 participants reporting ≥ 2 aura symptoms in succession, 39 (68.4%) identified visual aura as the initial symptom. This aligns with existing evidence and lends support to the hypothesis that cortical spreading depolarization (CSD) – the neurobiologic substrate of aura – commences in the occipital cortex, subsequently spreading in an anterior direction [25]. The reason for this is unclear, but it underlines the potential value of directing neuroimaging efforts towards the occipital region to ideally detect evidence of CSD.

### Headache sequence

In most of our participants (80.8%) reporting aura accompanied by headache, aura symptoms were reported to precede the onset of headache during a migraine attack. This is also a common description in the scientific literature, but caution is needed to avoid misdiagnosis. Indeed, our findings indicate that 1 in 5 persons with MA might experience aura symptoms after the onset of headache. The complex relationship between CSD and the headache phase of migraine remains enigmatic. It is yet to be ascertained whether CSD acts as a trigger for the headache phase or if an unidentified factor precipitates both CSD and headache. The fact that a substantial proportion of our participants experience headache either preceding or coinciding with aura onset challenges the conventional belief of CSD being the predominant causative agent. Interestingly, recent research demonstrated that intravenous CGRP infusion can induce migraine aura in individuals with migraine with aura. It is proposed that peripherally acting exogenous CGRP activates the trigeminovascular system which in turn trigger CSD and thereby aura symptoms. This mechanism could elucidate headache preceding or coinciding with aura [26]. The relationship between aura and headache remains an important area of research in the future.

### Headache features

Among our 227 participants with MA, 202 (89.0%) reported experiencing co-existing MO, leaving only 25 (11.0%) who had never experienced MO. The concurrent diagnosis of MA and MO in our study diverges from much of the existing literature, where the two are often treated as mutually-exclusive diagnoses [27–29]. In studies that do permit dual diagnoses, co-existence prevalence rates for MA and MO have been reported to be as low as 4% [30]. It therefore seems warranted for future population-based studies to determine the prevalence of co-existing MO and MA more appropriately. A better understanding this relationship might have both clinical and pathophysiologic implications.

### Migraine aura without headache

A subset of our participants experienced the phenomenon of migraine aura without headache. Of note, one-third recounted instances of this phenomenon, with some never associating a headache with their aura symptoms. A population-based study has previously found that 38% of people with MA have experienced aura without headache [9]. Yet, the phenomenon of migraine aura without headache remains poorly understood in epidemiologic research. It therefore seems warranted to advocate for independent evaluations of aura symptoms with and without a relation to headache. Delving into the experiences of patients who exclusively manifest migraine aura without headache could be instrumental in exploring the neurobiologic underpinnings of MA.

### Differential diagnoses and red flags

In clinical practice, an important differential diagnosis to MA is transient ischemic attacks, which can manifest with transient aura-like symptoms. A useful diagnostic heuristic is to view positive and gradually spreading symptoms as indicative of MA, while sudden onset and negative symptoms usually suggest an ischemic cause. Echoing this, almost all our participants (96.9%) experienced symptoms that were positive or gradually spreading over ≥ 5 min.

Another important aspect in clinical practice is the perception of side-locked aura (i.e., aura symptoms exclusively localized to one side) as a potential red flag, often prompting neuroimaging. Yet, the current guidelines from the American Headache Society do not specify whether neuroimaging is needed based on the presence of side-locked aura [31]. Among our participants, side-locked manifestations were reported by 23.2% with visual aura and 43.2% with somatosensory aura, underscoring its fairly frequent occurrence. Future studies should delve into whether neuroimaging holds diagnostic value in the context of side-locked aura.

### Strengths and limitations

Our study has several strengths, with the most important ones being the large sample size and the use of a semi-structured interview. The latter allows the flexibility for interviewers to rephrase questions and clarify participants’ responses, further bolstering the accuracy of our data. However, there are also some limitations that must be mentioned. The retrospective reporting of aura symptoms, without prospective recording, introduces potential recall bias. It is worth noting that discrepancies have been demonstrated between retrospective descriptions and prospective recordings of headache in relation to migraine aura [12]. Yet, our approach mirrors the conventional clinical scenario where diagnoses rely on patients' retrospective symptom narratives, enhancing the relevance of our findings to real-world clinical practice. Recall bias is particularly relevant for participants with infrequent aura, constituting 14.1% of our cohort. While our investigation provides valuable insights into typical aura presentation, we did not assess variability between individual aura episodes. Future prospective studies should address this aspect to offer a more comprehensive understanding of migraine with aura. We recorded the number of aura episodes in the past month, past 3 months, and past year, as well as the lifetime number of aura episodes. We did not, however, investigate possible fluctuations in aura frequency over time. This would be an interesting topic of investigation for future studies. Moreover, eligible participants were required to experience ≥ 4 monthly migraine days, and the majority of participants were recruited from a tertiary headache center. A high proportion of our participants had chronic migraine and/or medication-overuse headache. The generalizability of our findings might therefore not extend to those with less frequent migraine or patients in other health sectors.

## Conclusions

Among 227 adults diagnosed with MA, the predominant manifestation was visual aura, whilst somatosensory and speech and/or language were less frequent. Our intricate delineation of aura types, their sequences, and relation to the headache phase of migraine offers profound insights into the clinical manifestations of MA. These observations emphasize the importance of detailed clinical assessments and pinpoint avenues for future research, in particular focusing on the interplay between the aura and headache phase of migraine.

### Supplementary Information

**Additional file 1.**

## Data Availability

Upon reasonable request, the corresponding author will provide the necessary data and materials to interested researchers for the purpose of academic scrutiny, reproducibility, and further scientific investigation.

## Abbreviations

CSD: Cortical spreading depolarization
ICHD-3: The International Classification of Headache Disorders 3rd edition
MA: Migraine with aura
REFORM: The Registry for Migraine study
SD: Standard deviation
