# Supplementary material for: Clinical features of migraine with aura: a REFORM study
Source: J Headache Pain. 2024 Feb 13;25(1):22. doi: 10.1186/s10194-024-01718-1 (PMC10865578; doi:10.1186/s10194-024-01718-1)
Supplement: Supplementary file 1 — Additional file 1. [file 10194_2024_1718_MOESM1_ESM.docx]

**Supplemental Table S1: Comparisons of aura features between participants using or not using concomitant preventative medication for migraine.**

|  |  |  |  |
| --- | --- | --- | --- |
|  |  | Concomitant prophylactic medication | |
|  | **All (n = 227)** | **Yes (n = 119)** | **No (n = 108)** |
| **Visual aura** | 215 (94.7) | 116 (97.5) | 99 (88.9) |
| Positive symptoms | 192 (89.3) | 104 (89.7) | 88 (88.9) |
| Negative symptoms | 127 (59.1) | 71 (61.2) | 56 (56.6) |
| Zig-zag pattern | 76 (35.3) | 43 (37.1) | 33 (33.3) |
| Flickering | 151 (70.2) | 84 (72.4) | 67 (67.7) |
| Sawtooth edge | 30 (14.0) | 19 (16.4) | 11 (11.1) |
| Gradually spreading | 155 (72.1) | 84 (72.4) | 71 (71.7) |
| Central onset* | 45 (20.9) | 27 (23.3) | 18 (18.2) |
| Peripheral onset* | 109 (50.7) | 56 (48.3) | 53 (53.5) |
| Mean duration (min) | 49.2 (109.7) | 40.5 (56.1) | 59.4 (149.6) |
| Duration <5 minutes | 8 (3.7) | 4 (3.4) | 4 (4.0) |
| Duration 5-60 minutes | 185 (86.0) | 101 (87.1) | 84 (84.8) |
| Duration >60 minutes | 22 (10.2) | 11 (9.5) | 11 (11.1) |
| Unilateral symptoms | 109 (50.7) | 65 (56.0) | 44 (44.4) |
| Sidelocked symptoms** | 51 (23.7) | 33 (28.4) | 18 (18.2) |
| Aura ipsilateral to headache*** | 18 (8.4) | 10 (8.6) | 8 (8.1) |
| Aura contralateral to headache*** | 64 (29.8) | 38 (32.8) | 26 (26.3) |
| **Somatosensory aura** | 81 (35.7) | 39 (32.8) | 42 (38.9) |
| Positive symptoms | 69 (85.2) | 34 (87.2) | 35 (83.3) |
| Negative symptoms | 28 (34.6) | 13 (33.3) | 15 (35.7) |
| Gradually spreading | 58 (71.6) | 28 (71.8) | 30 (71.4) |
| Mean duration (min) | 136.7 (503.4) | 172.2 (691.8) | 103.8 (220.3) |
| Duration <5 minutes | 2 (2.5) | 1 (2.6) | 1 (2.4) |
| Duration 5-60 minutes | 56 (69.1) | 31 (79.5) | 25 (59.5) |
| Duration >60 minutes | 23 (28.4) | 7 (17.9) | 16 (38.1) |
| Unilateral symptoms | 55 (67.9) | 28 (71.8) | 27 (64.3) |
| Sidelocked symptoms** | 35 (43.2) | 18 (46.2) | 17 (40.5) |
| Aura ipsilateral to headache*** | 8 (9.9) | 5 (12.8) | 3 (7.1) |
| Aura contralateral to headache*** | 35 (43.2) | 15 (38.5) | 20 (47.6) |
| **Speech and/or language aura** | 31 (13.7) | 17 (14.3) | 14 (13.0) |
| Non-fluent aphasia | 23 (74.2) | 11 (64.7) | 12 (85.7) |
| Fluent aphasia | 5 (16.1) | 2 (11.8) | 3 (21.4) |
| Dysarthria | 9 (29.0) | 9 (52.9) | 0 (0) |
| Mean duration (min) | 65.7 (109.7) | 69.5 (138.4) | 60.8 (59.2) |
| Duration <5 minutes | 1 (3.2) | 1 (5.9) | 0 (0) |
| Duration 5-60 minutes | 24 (77.4) | 15 (88.2) | 9 (64.3) |
| Duration >60 minutes | 5 (16.1) | 1 (5.9) | 4 (28.6) |
| **Number of aura symptoms** |  |  |  |
| 1 | 148 (65.2) | 77 (64.7) | 71 (65.7) |
| 2 | 58 (25.6) | 31 (26.1) | 27 (25.0) |
| 3 | 20 (8.8) | 11 (9.2) | 9 (8.3) |
| 4 | 1 (0.4) | 0 | 1 (0.9) |
|  |  |  |  |

**Supplemental Table S1 legend:** Data are mean (SD) or n (%). *Includes only participants with gradually spreading symptoms. **Includes only participants with unilateral symptoms. ***Includes only participants with both unilateral aura symptoms and unilateral headache. Abbreviations: SD: Standard deviation.
